# Mild Cognitive Impairment Predicts Institutionalization among Older Men: A Population-Based Cohort Study

**DOI:** 10.1371/journal.pone.0046061

**Published:** 2012-09-24

**Authors:** Danijela Gnjidic, Fiona F. Stanaway, Robert Cumming, Louise Waite, Fiona Blyth, Vasi Naganathan, David J. Handelsman, David G. Le Couteur

**Affiliations:** 1 Departments of Clinical Pharmacology and Aged Care, Royal North Shore Hospital, Sydney, New South Wales, Australia; 2 Sydney Medical School, University of Sydney, Sydney, New South Wales, Australia; 3 Centre for Education and Research on Ageing, Concord Hospital, Sydney, New South Wales, Australia; 4 ANZAC Research Institute, Concord Hospital, Sydney, New South Wales, Australia; 5 Sydney School of Public Health, University of Sydney, Sydney New South Wales, Australia; 6 Faculty of Pharmacy, University of Sydney, Sydney, New South Wales, Australia; Cardiff University, United Kingdom

## Abstract

**Background:**

There is a lack of evidence on the contribution of mild cognitive impairment (MCI) to institutionalization in older adults. This study aimed to evaluate a range of risk factors including MCI of institutionalization in older men.

**Methods:**

Men aged ≥70 years (n = 1705), participating in the Concord Health and Ageing in Men Project, Sydney, Australia were studied. Participants completed self-reported questionnaires and underwent comprehensive clinical assessments during 2005–2007. Institutionalization was defined as entry into a nursing home facility or hostel at any time over an average of 5 years of follow-up. Cox regression analysis was conducted to generate hazard ratios (HR) with 95% confidence intervals (CI).

**Results:**

A total of 125 (7.3%) participants were institutionalized. Piecewise Cox proportional models were generated and divided at 3.4 years (1250 days) of follow-up due to violation of the proportional hazards assumption for the association between MCI and institutionalization (χ^2^ = 6.44, p = 0.01). Dementia, disability in Activities of Daily Living (ADL) and Instrumental Activities of Daily Living (IADL), poor grip strength, few social interactions, being a Non-English speaking immigrant and age were predictive of institutionalization during both time periods, whereas MCI (HR = 4.39, 95%CI 2.17–8.87) only predicted institutionalization in the period beyond 3.4 years of follow-up. Being married (HR = 0.42, 95%CI: 0.24–0.72) was protective only during the period after 3.4 years of follow-up.

**Discussion:**

In this study, the strongest predictors of institutionalization were dementia, MCI, ADL and IADL disability. MCI was not a predictor of early institutionalization but became a significant predictor beyond 3.4 years of follow-up.

## Introduction

Delaying older adults’ transition from living in the community to institutionalization is of major public health importance. It is also important for older individuals themselves, most of whom would prefer to remain living in the community [1]. A number of studies, conducted across a range of settings (eg. population-based and dementia-based samples) have investigated risk factors for institutionalization in older adults [1], [2]. Dementia, disability in Activities of Daily Living (ADL) and Instrumental Activities of Daily Living (IADL) are the most consistent risk factors for admission to a residential aged care facility (RACF) [1], [2]. However, other studies have identified sociodemographic and socioeconomic factors as additional important predictors of institutionalization in older adults [3], [4].

While most studies that have investigated predictors of institutionalization have looked at severe cognitive impairment, the contribution of mild cognitive impairment (MCI) to institutionalization in older adults is not clear. One study has looked at the association of cognitive impairment not including dementia with adverse outcomes including institutionalization in older adults [5]. However, the diagnosis was based on clinical judgment rather than the use of specific diagnostic criteria.

More research is also needed to understand factors contributing to institutionalization in an ethnically diverse population. There is growing ethnic diversity in older populations in many western countries including Australia, Canada, and the USA [6], [7], [8]. Cultural differences in values and expectations of family support as well as the availability of culturally appropriate residential aged care services could all contribute to different rates of institutionalization for minority elders.

To our knowledge, no study has been conducted to investigate MCI as a risk factor of institutionalization in older adults. The objective of this study was to investigate a range of risk factors including demographics, socioeconomic status, health risk factors, health conditions including MCI, physical performance, medication use and service use as predictors of institutionalization in an ethnically diverse community-based cohort of older men, enrolled in the Concord Health and Ageing in Men Project (CHAMP). Investigating predictors of institutionalization in the CHAMP cohort represents a unique opportunity due to the availability of data including a range of clinical assessments, cognitive assessments, physical performance measures and use of community-based home care services.

## Methods

### Study Population

Participants were community-dwelling older men, participating in the CHAMP study, an ongoing cohort study in Sydney, Australia [9]. Eligible participants were aged ≥70 years at baseline and living in a specific study area (the Local Government Areas of Burwood, Canada Bay and Strathfield) near Concord Hospital. The only exclusion criterion was living in a RACF. The Electoral Roll was chosen as the sampling frame for the study. Registration on the Electoral Roll is compulsory and regularly updated, making it a suitable population-wide sampling frame. Invitation letters were sent to 3627 men and contact was made with 3005. Most of the 622 men who were not contacted did not have a listed telephone number. One hundred and ninety of the contacted men were not eligible for the study because they had moved out of the study area, moved into a nursing home, or had died. Of the 2815 eligible men contacted, 1511 (53.7%) participated in the study. An additional 194 (11.4%) men living in the study area heard about the study from friends or the local media and were recruited before receiving an invitation letter, giving a final sample of 1705 participants. All participants gave written informed consent. The study was approved by the Human Research Ethics Committee Concord RG Hospital.

### Data Collection

Participants underwent baseline assessments that comprised self-completed study questionnaires and a clinical assessment that consisted of physical performance measures, neuropsychological testing and medication inventory. Following the initial baseline assessment, the men were contacted regularly at 4-monthly intervals to enable updating of data on institutionalization. Data collected during baseline assessments (2005–7), including self-reported questionnaire data and clinical information, were used in the current analysis along with longitudinal data on institutionalization.

### Ascertainment of Predictor Variables

The main groups of predictor variables included demographic factors, socioeconomic status, health risk factors, health conditions, physical performance measures, medication use and service use. These predictors have been identified based on the clinical significance, and based on previous studies investigating risk factors for institutionalization [1], [2].

#### Sociodemographic factors

Sociodemographic variables included age, marital status (married versus other) and living arrangements (live alone versus live with others). Social support was measured using the shortened Duke Social Support Index (DSSI) which measures both social support satisfaction and social interactions [10]. The first item in the DSSI was modified in the CHAMP which allowed the creation of two separate variables for the number of family and non-family supports. These variables were entered into models separately to the score for social interactions and subjective support. The men were also asked their country of birth which enabled grouping of the men into the categories of Australian-born, overseas-born from an English-speaking country (ESB), and overseas-born from a non-ESB. The CHAMP study area has a high proportion of immigrants and as a result, only 49.8% of men in the CHAMP study were born in Australia and 19.6% were born in Italy.

#### Socioeconomic status

Socioeconomic status was measured using four separate variables: age at leaving school, main lifetime occupation (managers and professionals versus other), source of income (government pension only versus other) and house ownership.

#### Health risk factors

Physical activity was assessed using the Physical Activity Scale for the Elderly (PASE) [11]. Participants were asked about whether they had ever consumed alcohol and whether they had consumed at least 12 alcoholic drinks in the past 12 months. This enabled categorization of current non-drinkers into lifelong abstainers and ex-drinkers. For those who consumed at least 12 drinks in the past year, the frequency and quantity of alcohol consumption was assessed, enabling categorization of drinkers as either safe drinkers (1–21 drinks per week) or harmful drinkers (>21 drinks per week) [12]. Smoking status (never smoker, ex-smoker, current smoker) was also assessed.

#### Health conditions

Data on medical conditions were obtained from the self-reported questionnaires in which participants reported if they had any of the following diseases: diabetes, thyroid dysfunction, osteoporosis, Paget’s disease, stroke, Parkinson’s disease, epilepsy, hypertension, coronary artery disease or myocardial infarction, angina, congestive heart failure, intermittent claudication, chronic obstructive lung disease, liver disease, chronic kidney (renal) disease or kidney (renal) failure, cancer (excluding non-melanoma skin cancers), or arthritis. The number of reported comorbidities was dichotomized at the upper quartile (≤4 versus>4).

Participants also self-reported the presence of shortness of breath, and a history of having fallen in the past 12 months. Data on self-rated health were obtained and dichotomized into excellent/good versus fair/poor/very poor. Corrected visual acuity was assessed using a Bailey-Lovie chart [13] and poor vision was defined as those with <6/19 visual acuity. The presence of incontinence was defined as leaking urine at least two or three times a week. Participants were asked about the presence of chronic pain (pain in the last six months that has lasted for ≥3 months and been experienced every day). Participants were also asked how much pain interfered in their normal activities in the past four weeks as part of the Short Form 12 [14]. Participants were considered to have chronic intrusive pain if they reported the presence of chronic pain and pain that interfered with normal activities moderately, quite a bit or extremely. Depressive symptoms were assessed with the 15-item Geriatric Depression Scale (≥5 indicative of depressive symptoms) [15]. Anxiety symptoms were measured using the Goldberg Anxiety Scale [16], with >5 considered as presence of anxiety.

#### Diagnosis of cognitive impairment

Participants were screened for cognitive impairment using the Mini Mental State Examination (MMSE) [17] and the Informant Questionnaire on Cognitive Decline (IQCODE) [18] during the baseline clinic assessment. In addition to the cognitive screen participants also completed other cognitive assessments including Addenbrooke’s Cognitive Examination [19], Trail Making Task B [20], Weigl-Colour Form Sorting test [21] and Logical Memory Recall test [22]. Participants with a MMSE less than or equal to 26 and/or IQCODE greater than 3.6 were invited to have detailed clinical assessments by the study geriatrician. This assessment included a review of medical comorbidities and medications, a standardized neurological assessment, a more detailed informant interview [23] and the Rowland Universal Dementia Assessment Scale (RUDAS) [24]. At a weekly consensus meeting two geriatricians, a neurologist and a neuropsychologist reviewed all medical, cognitive, informant and functional data and reached a final diagnosis of cognitive status for each participant. At the end of the screening and clinical assessments, participants were categorized as having dementia (n = 93), MCI (n = 120), unknown cognitive status (n = 164) or cognitively intact (n = 1328). Participants determined to be cognitively impaired but not demented were given the diagnosis of MCI, if they met the clinical criteria described by Petersen et al 2004 [25]. Although MCI was categorized according to the sub-types defined by Petersen et al, for the purposes of analyses and given small cell sizes, participants with all sub-types of MCI were grouped together. This is consistent with subjects fulfilling the general criteria for MCI [26]. Diagnosis and classification of dementia was based on the Diagnostic and Statistical Manual of Mental Disorders (4^th^ edition) revised criteria and well recognized criteria for dementia subtypes [27], [28], [29]. All sub-types of dementia were grouped together for analyses given small cell sizes.

#### Physical function and performance

Functional status was measured with ADL and IADL scales. Disability in ADL was defined as needing help with ≥1 activities included in the modified Katz ADL scale [30]. Disability in IADL was defined as needing help with ≥1 activities included in the OARS IADL scale [31]. Physical performance was assessed by administering a standard performance battery that included the following tasks: (i) walking speed (m/s) over a 6-m course, adjusted for height; (ii) chair stands test-time to successfully complete five chair stands was assessed and time dichotomized at the slowest quartile; (iii) muscle (grip) strength (kg), and (iv) dynamic balance test. Muscle strength was measured using a Jamar dynamometer (Promedics, Blackburn, UK). The score was calculated as the grip strength (kg) of the dominant hand (best of two trials). Dynamic balance was assessed with a coordinated stability task [32]. Scores were dichotomized at the highest (worst) quartile. Participants, who did not complete the tests due to physical inability, were included in the worst quartile for the corresponding performance measures.

#### Medication assessment

Medication data were coded using the Iowa Drug Information Service (IDIS) drug code numbers. Polypharmacy was defined as the use of ≥5 regular prescription medicines [33]. Psychotropic medication use was defined as exposure to the following drug classes: anticonvulsants (IDIS code level 28120000), antidepressants and antipsychotics (IDIS code level 28160000) and anxiolytics (IDIS code level 28240000).

#### Service use variables

Participants were asked about their use of a number of community services during the past 12 months. These services included: spending at least one day in an aged care day centre, being visited by Home Care to help with personal or household duties, using services of the Community and Aged Care Packages (CACPs), or any service to deliver or prepare meals at home. Participants were categorized as using one or more of these services in the past year versus using none of these services. Participants were also asked whether they had spent at least one night in a hostel or nursing home in the past 12 months and this was entered into models as a separate variable.

### Ascertainment of Outcome Variable

Institutionalization was defined as entry into a nursing home facility or hostel at any time during follow-up to 6.58 years. In Australia, there are two main forms of residential aged-care facilities: low-level care facilities (hostels) and high-level care facilities (nursing homes). Self-care retirement villages are not considered to be RACF and so moving into one of these facilities was not considered “institutionalization”. Data on institutionalization were ascertained through a regular phone contact with the participants or their nominated contact person at 4-monthly intervals. While our data does not enable us to discriminate between permanent and respite institutionalization, the majority of admission to aged-care facilities in Australia are permanent [34].

### Statistical Analysis

Data are summarized as means (standard deviation) or numbers (proportions). Differences between institutionalized and non-institutionalized participants were compared using the two-sided t-test or χ^2^-test where appropriate. Initial univariate analyses of the association between the various study measures and institutionalization were conducted using Log-rank tests and examination of survival curves. Tests for linear trends were performed for continuous variables to determine the linearity of their relationship with institutionalization and, hence, whether to enter these variables into models as continuous or categorical variables. Testing for co-linearity between the variables was performed. There was no evidence of correlation between the variables. The appropriate parameterization of continuous variables as either categorical or continuous was also confirmed in the final model by using Akaike’s Information Criterion (AIC). Univariate Cox regressions were conducted to determine unadjusted hazard ratios for admission to an aged care facility for the various study measures. Variables that had a *p*<0.25 in univariate analyses were included in the multivariate model with institutionalization as the outcome. Backward stepwise elimination was used to eliminate non-significant variables from the multivariate model. Backward stepwise elimination has an advantage over other methods (eg. forward) as it allows to examine a model with all independent variables as well as the joint predictive capability of all variables. Clinically significant interactions, and interactions identified in previous studies between dementia and urinary incontinence, dementia and falls, and arthritis and pain were examined by adding the interaction terms into the main effect models one at a time and including the significant interaction terms in the final model. None of the interaction terms remained significant in the model. In the final model, the proportional hazards assumption was assessed through use of a time-dependent covariate method, analysis of Schoenfeld residuals plots and graphical methods (eg. survival plots) for each variable. Upon the examination of the results of time-dependent covariate method, Schoenfeld residuals plots and survival curves, it was identified that the MCI covariate was violating the assumptions. To address this, the step function proportional hazards or piecewise Cox model was used to test the effect of MCI on institutionalization. Data were analyzed using SAS version 9.2 (SAS Institute Inc., Cary, North Carolina). The Kaplan-Meier survival curves were generated using SPSS software version 19.0 (SPPS Inc, Chicago, Illinois).

## Results

**Figure 1:**
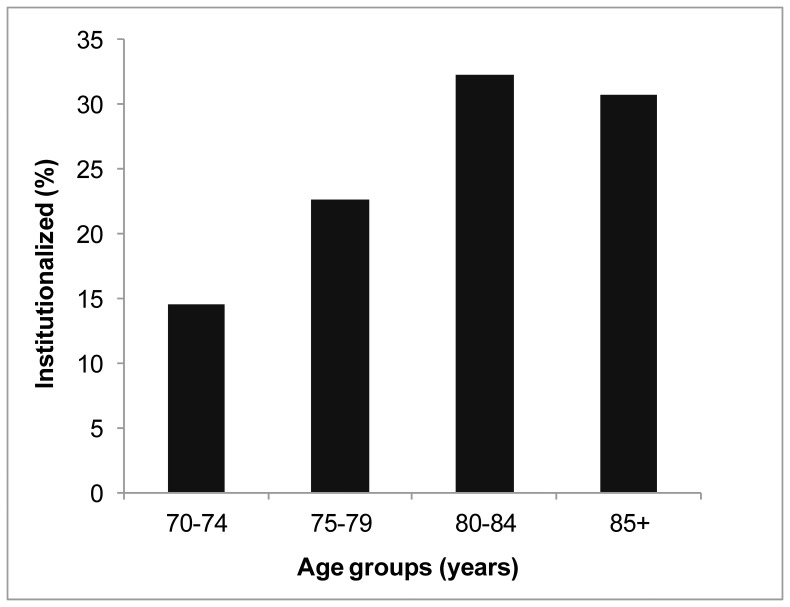
The percentage of participants institutionalized with increasing age. Test for deviation from linear trend: P = 0.0003.

Descriptive characteristics are provided in Table 1. Of 1705 men studied at baseline, a total of 125 (7.3%) were institutionalized during a mean follow-up of 4.94 (range: 0.08–6.58) years. The mean age, social support satisfaction and social interactions were significantly different between institutionalized men compared with non-institutionalized men. The proportion of men institutionalized increased up to the age of 84 years, and then slightly dropped (Figure 1). In relation to health conditions, there were significant differences in all factors apart from the presence of anxiety symptoms between the two groups.

**Table 1:** Characteristics of the study population according to institutionalization status.

Variable	Total population (n = 1705)^a^	Institutionalized(n = 124, 7.3%)	Not institutionalized(n = 1581, 92.7%)	P values
***Socio-demographic factors***				
Age, mean (SD)	76.9 (5.5)	81.4 (5.7%)	76.6 (5.3%)	<0.001
Currently married	1278 (74.9%)	73 (58.9%)	1205 (76.2%)	<0.001
Live alone	318 (18.1%)	44 (35.5%)	274 (17.5%)	<0.001
Social support satisfaction, high (DSSS score ≥19)	1294 (76.9%)	69 (57.0%)	1225 (78.5%)	<0.001
Social interactions, high (DSSS score ≥9)	1019 (61.2%)	48 (40.3%)	971 (62.8%)	<0.001
Country of birth				
Australia	849 (49.8%)	82 (66.1%)	767 (48.5%)	
ESB immigrant	105 (6.2%)	9 (9.9%)	96 (11.2%)	
Non-ESB immigrant	751 (44.1%)	33 (28.7%)	718 (48.4%)	0.0002
***Socio-economic factors***				
Occupation, professional	505 (29.8%)	21 (16.9%)	484 (30.8%)	0.0011
Own house outright	1494 (88.9%)	104 (86.0%)	1390 (89.2%)	0.27
Years of education, ≥7 years	1429 (84.7%)	115 (94.3%)	1314 (83.9%)	0.002
Source of income, pension only	773 (45.9%)	66 (54.1%)	707 (45.3%)	0.06
***Health risk factors***				
Physical activity, normal/high (PASE score ≥80)	1263 (74.9%)	55 (45.5%)	1208 (77.2%)	<0.001
Alcohol consumption				
Lifelong non-drinker	147 (8.8%)	8 (6.5%)	139 (9.0%)	
Ex-drinker	250 (14.9%)	30 (24.4%)	220 (14.2%)	
Safe drinker (1–21 drinks per week)	1151 (68.7%)	77 (62.6%)	1074 (69.2%)	
Harmful drinker (>21 drinks per week)	127 (7.6%)	8 (6.5%)	119 (7.7%)	0.02
Smoking status				
Never smoker	629 (37.3%)	51 (41.8%)	578 (37.0%)	
Previous smoker	956 (56.7%)	62 (50.8%)	894 (57.2%)	
Current smoker	101 (6.0%)	9 (7.4%)	92 (5.9%)	0.38
***Health conditions***				
Comorbidities, ≥5	237 (14.0%)	30 (24.6%)	207 (13.2%)	0.0005
Urinary incontinence	232 (14.0%)	27 (23.3%)	205 (13.3%)	0.003
Visual acuity, low (<6/19)	74 (4.5%)	15 (12.5%)	59 (3.9%)	<0.001
Chronic intrusive pain	223 (13.4%)	24 (20.5%)	199 (12.8%)	0.02
Self-rated health, good or excellent	1176 (69.9%)	68 (57.1%)	1108 (70.9%)	0.002
Shortness of breath	210 (13.3%)	24 (19.4%)	186 (11.8%)	0.013
Hearing loss	1027 (61.1%)	85 (70.3%)	942 (60.4%)	0.03
History of falls	322 (19.1%)	50 (41.7%)	272 (17.4%)	<0.001
Depressive symptoms	246 (14.6%)	45 (37.5%)	201 (12.9%)	<0.001
Anxiety symptoms	123 (7.4%)	10 (8.7%)	113 (7.3%)	0.57
Cognitive status				
Normal	1492 (87.5%)	73 (58.9%)	1419 (89.8%)	
Mild cognitive impairment	120 (7.0%)	14 (11.3%)	106 (6.7%)	
Dementia	93 (5.5%)	37 (29.8%)	56 (3.5%)	<0.001
***Physical function and performance***				
ADL disability (needing help with ≥1 task)	141 (8.3%)	40 (32.3%)	101 (6.4%)	<0.001
IADL disability (needing help with ≥1 task)	697 (41.6%)	97 (80.8%)	600 (38.6%)	<0.001
Grip strength, poor (lowest quartile and unable)	486 (28.7%)	71 (58.2%)	415 (26.4%)	<0.001
Chair stands, slow (lowest quartile and unable)	462 (27.7%)	74 (63.3%)	388 (25.0%)	<0.001
Walking speed, slow (lowest quartile and unable)	242 (14.5%)	59 (49.6%)	183 (11.8%)	<0.001
Dynamic balance test, poor (lowest quartile and unable)	478 (29.1%)	70 (60.3%)	408 (26.7%)	<0.001
***Medication use***				
Polypharmacy (≥5 medicines)	639 (37.7%)	52 (42.3%)	587 (37.3%)	0.27
Psychotropic medications	211 (12.4%)	22 (17.9%)	189 (12.0%)	0.06
***Service use***				
Stay in nursing home in past year	29 (1.7%)	9 (7.4%)	20 (1.3%)	<0.001
Use of services in past year	193 (11.3%)	49 (39.5%)	144 (9.1%)	<0.001

ADL = Activities of Daily Living [30]; DSSI = Duke Social Support Index [10]; IADL = Instrumental Activities of Daily Living [31]; PASE = Physical Activity Scale for the Elderly [11].

a Missing data not included in percentages.

**Figure 2:**
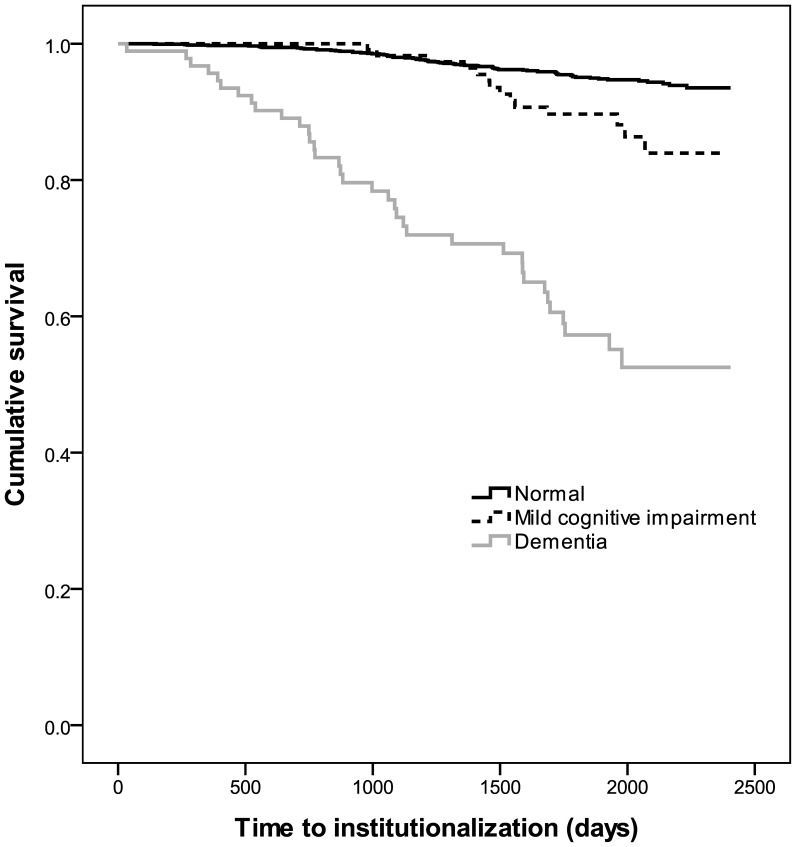
Kaplan-Meier survival curves of the time until institutionalization by cognitive status groups.

The multivariate Cox proportional model showed that age, marital status, social satisfaction, social interactions, country of birth, alcohol use, cognitive status, ADL disability, IADL disability, grip strength, and service use were significant predictors of institutionalization. However, the use of a time-dependent covariate and analysis of Schoenfeld residuals demonstrated that MCI violated the proportional hazards assumption (χ^2^ = 6.44, p = 0.01). Therefore, as the effect of MCI on institutionalization was not stable over entire follow-up time, and the proportional hazards assumption was not valid, the piecewise Cox proportional models were used to test the effect of MCI on institutionalization. The follow-up period was divided at 3.4 years (1250 days) based on examination of the survival curve for MCI and institutionalization (Figure 2).

Table 2 shows the results of the Cox proportional hazards models for the first 3.4 years of follow-up (Model 1) and beyond 3.4 years (Model 2) of follow-up. In the Cox regression model up to 3.4 years of follow-up, age, high social interactions, country of birth, dementia, ADL disability, IADL disability, grip strength and service use were significant predictors of institutionalization. Dementia (HR = 5.43, 95%CI: 3.00–9.81), ADL disability (HR = 3.22, 95%CI: 1.80–5.77), IADL disability (HR = 3.01, 95%CI: 1.32–6.86) and use of services (HR = 2.61, 95%CI: 1.46–4.66) were the most significant predictors of institutionalization. Interestingly, MCI was not associated with institutionalization during this time interval (HR = 0.72, 95%CI: 0.22–2.36). Participants were less likely to be institutionalized if they had high social interactions (HR = 0.48, 95%CI: 0.27–0.85) and were a NESB immigrant (HR = 0.31, 95%CI: 0.16–0.60).

**Table 2:** Final multivariate models for predictors of institutionalization up to 3.4 years and beyond 3.4 years of follow-up.

	Model 1: Predictors up to 3.4 years (n = 1658)		Model 2: Predictors beyond 3.4 years (n = 1428)	
Predictors	Hazard Ratio(95% confidence interval)	P value	Hazard Ratio(95% confidence interval)	P value
Age group (80+ vs <80)	1.90 (1.05–3.45)	0.04	2.37 (1.37–4.08)	0.002
Marital status (currently married vs not married)	NA	NA	0.42 (0.24–0.72)	0.002
Social Interactions (high vs low)	0.48 (0.27–0.85)	0.01	0.47 (0.26–0.87)	0.02
ESB immigrant vs Australian born	0.91 (0.35–2.37)	0.85	0.92 (0.32–2.61)	0.87
NESB immigrant vs Australian born	0.31 (0.16–0.60)	0.0005	0.41 (0.22–0.77)	0.005
MCI vs normal	0.72 (0.22–2.36)	0.58	4.39 (2.17–8.87)	<0.001
Dementia vs normal	5.43 (3.00–9.81)	<0.001	6.05 (2.95–12.44)	<0.001
ADL disability (yes vs no)	3.22 (1.80–5.77)	<0.001	2.72 (1.36–5.46)	0.005
IADL disability (yes vs no)	3.01 (1.32–6.86)	0.009	2.71 (1.43–5.13)	0.002
Grip strength (low vs high)	2.19 (1.22–3.93)	0.008	1.95 (1.14–5.13)	0.002
Service use (yes vs no)	2.61 (1.46–4.66)	0.001	NA	NA

ADL = Activities of Daily Living [30]; ESB = English Speaking Background; IADL = Instrumental Activities of Daily Living [31];

MCI = Mild Cognitive Impairment; NESB = Non-English Speaking Background.

NA, not applicable = not a significant predictor during the time period.

In the Cox regression model for the period beyond 3.4 years of follow-up, age, marital status, social interactions, country of birth, cognitive status, ADL disability, IADL disability, and grip strength were statistically significant predictors of institutionalization. Dementia (HR = 6.05, 95%CI: 2.95–12.44), MCI (HR = 4.39, 95%CI: 2.17–8.87), ADL disability (HR = 2.72, 95%CI: 1.36–5.46) and IADL disability (HR = 2.71, 95%CI: 1.43–5.13) were the most significant predictor factors for institutionalization in this later time period. Married participants (HR = 0.42, 95%CI: 0.24–0.70), NESB immigrants (HR = 0.41, 95%CI: 0.22–0.77), and those with high social interactions (HR = 0.47, 95%CI: 0.26–0.87) were less likely to be institutionalized.

## Discussion

In this prospective population-based study, we identified a number of predictors of institutionalization. The strongest predictors were dementia, MCI, ADL and IADL disability. Older adults with dementia had approximately six times the risk of institutionalization compared with those who did not have dementia. The predictive value of MCI changed with the length of follow-up. MCI was a significant predictor of institutionalization beyond 3.4 years of follow-up. In this period, participants with MCI had approximately four times the risk of institutionalization compared with those who were cognitively intact.

The rate of institutionalization of 7.3% in this study is slightly lower when compared to previous studies conducted in population-based settings in Australia, Europe and USA. An Australian study of community-dwelling adults aged ≥60 years, reported an 8.7% permanent nursing home placement over 14 years of follow-up [35]. In a study of adults aged ≥75 years living in Germany, 7.8% of participants were institutionalized during a mean follow-up of 7.6 years [36]. A USA study reported an institutionalization rate of 13.6% over 12 years of follow-up [37]. The difference in the institutionalization rate across studies may be due to the greater ethnic diversity in the CHAMP study. Moreover, older men are generally less likely to be institutionalized as their wives commonly act as their caregiver at home.

The findings of this study are consistent with meta-analyses that have highlighted dementia, ADL and IADL disability as the most important predictors of institutionalization in older adults [1], [2], [36]. However, our study is the first study to show that MCI is an important predictor of institutionalization. Individuals with MCI are at an increased risk of developing dementia [38], [39]. Therefore, our finding that MCI only contributes to an increased risk of institutionalization after more than three years suggests that this increased risk is associated with a progression of the MCI to dementia with an associated increased risk of institutionalization. In the CHAMP population, of the 120 men diagnosed with MCI at baseline, 82 men were re-assessed at Year 2 follow-up. Of these, 12% (n = 10) had progressed to dementia over two years and three of these had been institutionalized. This progression rate of MCI to dementia is similar to other community-based studies [40]. However, other studies have reported higher conversion rates [41].

The prevalence of MCI has been found to be higher amongst older men than women in those living in nursing and veteran care homes [42]. Cognitive impairment, excluding dementia, has been shown to predict adverse outcomes including institutionalization and mortality in older adults [5]. Therefore, delaying the onset of dementia in individuals with MCI may reduce the risk of institutionalization, which is of major clinical and public health importance [43]. A recent study highlighted that not all predictors of institutionalization are robust with varying follow-up periods [44]. Identification of risk factors that predict institutionalization over short versus a longer period of time may inform future interventions to delay institutionalization [44].

We also found a strong relationship between country of birth, and risk of institutionalization. NESB immigrant men were about 70% less likely to be institutionalized compared with both Australian-born men. Different rates of institutionalization between ethnic groups has important implications for the planning of community and RACF services for older people, particularly as the proportion of older persons from an NESB in Australia is increasing [6]. Further research is required to confirm whether this difference in rates of admission is due to different cultural values about the role of family in supporting older persons to remain in the community or whether it is due to a relative lack of culturally and linguistically appropriate residential aged care services. Consistent with previous work [45], [46], we also found that being married was associated with a reduced risk of institutionalization.

Poor grip strength was also an important risk factor for institutionalization. One study has reported an association of weaker grip strength with increased risk of long-term nursing home stay in the unadjusted models only [47]. Interestingly, falls were not associated with an increased risk of institutionalization in our study, which is in contrast to previous studies [48], [49]. Urinary incontinence was also not a significant predictor of institutionalization in this population, which is consistent with one study [50] but not with another [51] conducted in community-dwelling older people. It may be that factors such as the physical performance measures and urinary incontinence are not significant predictors of institutionalization in models that already include ADL and IADL disability as these composite measures of function are determined in part by physical function and continence status.

The major strengths of the CHAMP study include its representative sampling from the community, detailed assessment of cognitive status, comprehensive, objective and clinically validated physical performance measures, and availability of a range of important risk factors for institutionalization. However, there are some limitations to the present study. We were unable to investigate the association of caregiver characteristics with the onset of institutionalization. Some studies have shown that in addition to participant characteristics, caregiver characteristics are important determinants of nursing home placement for persons with dementia [4], [52] while another study found that compared to participant characteristics, caregiver characteristics may not play an important role in predicting institutionalization [53]. This was a study of community-dwelling older men living in a defined geographical location, which may limit the study’s generalizabilty to men living in other areas. It should be noted that the response rate in the CHAMP study is similar to other comparable cohort studies of this type [9]. Also the findings of this study may not be applicable to older women. Moreover, the validity of self-report data in participants with cognitive impairment may be questionable.

In conclusion, in this population, the strongest predictors of institutionalization were dementia, MCI, ADL and IADL disability. Older adults with dementia had approximately a six times higher risk of institutionalization compared with those who did not have dementia. The contribution of MCI to institutionalization changed with time, with MCI being a significant predictor only beyond 3.4 years of follow-up. Participants with MCI had approximately a four times higher risk of institutionalization compared with those who were cognitively intact. Our findings suggest that in addition to other risk factors, MCI should also be considered when estimating the risks of long term institutionalization in older adults. Delaying the onset of dementia in individuals with MCI may reduce the risk of institutionalization in older adults, which is of major clinical and public health importance.

## References

[1] Luppa M, Luck T, Weyerer S, Konig HH, Brahler E, et al (2010) Prediction of institutionalization in the elderly. A systematic review. Age Ageing 39: 31–38. 19934075 10.1093/ageing/afp202

[2] Gaugler JE, Duval S, Anderson KA, Kane RL (2007) Predicting nursing home admission in the U.S: a meta-analysis. BMC Geriatr 7: 13. 17578574 10.1186/1471-2318-7-13 PMC1914346

[3] Heyman A, Peterson B, Fillenbaum G, Pieper C (1997) Predictors of time to institutionalization of patients with Alzheimer’s disease: the CERAD experience, part XVII. Neurology 48: 1304–1309. 9153462 10.1212/wnl.48.5.1304

[4] Lieberman MA, Kramer JH (1991) Factors affecting decisions to institutionalize demented elderly. The Gerontologist 31: 371–374. 1879712 10.1093/geront/31.3.371

[5] Tuokko H, Frerichs R, Graham J, Rockwood K, Kristjansson B, et al (2003) Five-year follow-up of cognitive impairment with no dementia. Arch Neurol 60: 577–582. 12707072 10.1001/archneur.60.4.577

[6] Gibson D, Braun P, Benham C, Mason F (2001) Projections of older immigrants: People from culturally and linguistically diverse backgrounds, 1996–2026. (AIHW cat no AGE 18) Canberra, Australian Capital Territory: Australia Institute of Health and Welfare.

[7] Turcotte M, Schellenberg G (2007) A portrait of seniors in Canada 2006 (Cat no. 89–519-XIE). Ottawa, Ontario: Statistics Canada.

[8] He W, Sengupta M, Velkoff VA, DeBarros KA (2005) 65+ in the United States: 2005. U.S. Census Bureau, Current Population Reports. Washington, DC: U.S. Government Printing Office.

[9] Cumming RG, Handelsman D, Seibel MJ, Creasey H, Sambrook P, et al (2009) Cohort Profile: the Concord Health and Ageing in Men Project (CHAMP). Int J Epidemiol 38: 374–378. 18480109 10.1093/ije/dyn071

[10] Koenig HG, Westlund RE, George LK, Hughes DC, Blazer DG, et al (1993) Abbreviating the Duke Social Support Index for use in chronically ill elderly individuals. Psychosomatics 34: 61–69. 8426892 10.1016/S0033-3182(93)71928-3

[11] Washburn RA, Smith KW, Jette AM, Janney CA (1993) The Physical Activity Scale for the Elderly (PASE): development and evaluation. J Clin Epidemiol 46: 153–162. 8437031 10.1016/0895-4356(93)90053-4

[12] Australian Institute of Health and Welfare (AIHW) (2011) Measuring alcohol risk in the 2010 National Drug Strategy Household Survey: implementation of the 2009 Alcohol Guidelines. Drug statistics series no. 26. Cat. no. PHE 152. Canberra.

[13] Bailey IL, Lovie JE (1976) New design principles for visual acuity letter charts. Am J Optom Physiol Opt 53: 740–745. 998716 10.1097/00006324-197611000-00006

[14] Ware JE, Kosinski M, Keller SD (1996) A 12-item short-form health survey: construction of scales and preliminary tests of reliability and validity. Med Care 34: 220–233. 8628042 10.1097/00005650-199603000-00003

[15] Yesavage JA, Brink TL, Rose TL, Lum O, Huang V, et al (1982) Development and validation of a geriatric depression screening scale: a preliminary report. J Psychiatr Res 17: 37–49. 7183759 10.1016/0022-3956(82)90033-4

[16] Goldberg D, Bridges K, Duncan-Jones P, Grayson D (1988) Detecting anxiety and depression in general medical settings. BMJ 297: 897–899. 3140969 10.1136/bmj.297.6653.897 PMC1834427

[17] Folstein MF, Folstein SE, McHugh PR (1975) “Mini-mental state”. A practical method for grading the cognitive state of patients for the clinician. J Psychiatr Res 12: 189–198. 1202204 10.1016/0022-3956(75)90026-6

[18] Jorm AF (1994) A short form of the Informant Questionnaire on Cognitive Decline in the Elderly (IQCODE): development and cross-validation. Psychol Med 24: 145–153. 8208879 10.1017/s003329170002691x

[19] Dudas RB, Berrios GE, Hodges JR (2005) The Addenbrooke’s cognitive examination (ACE) in the differential diagnosis of early dementias versus affective disorder. Am J Geriat Psychiatry 13: 218–226. 10.1176/appi.ajgp.13.3.218 15728753

[20] Reitan RM (1958) Validity of the Trail Making test as an indicator of organic brain damage. Percept Motor Skills 8: 271–276.

[21] Byrne LM, Bucks RS, Cuerden JM (1998) Validation of a new scoring system for the Weigl Color Form Sorting Test in a memory disorders clinic sample. J Clin Exp Neuropsychol 20: 286–292. 9777483 10.1076/jcen.20.2.286.1176

[22] Wechsler D (1987) Wechsler Memory Scale - Revised manual. San Antonio: The Psychological Corporation.

[23] Waite L, Grayson D, Jorm AF, Creasey H, Cullen J, et al (1999) Informant-based staging of dementia using the clinical dementia rating. Alzheimer Dis Assoc Disord 13: 34–37. 10192640 10.1097/00002093-199903000-00005

[24] Rowland JT, Basic D, Storey JE, Conforti DA (2004) The Rowland Universal Dementia Assessment Scale (RUDAS) and the Folstein MMSE in a multicultural cohort of elderly persons. Int Psychogeriatr 118: 111–120. 10.1017/S1041610205003133 16466591

[25] Petersen RC (2004) Mild cognitive impairment as a diagnostic entity. J Intern Med 256: 183–194. 15324362 10.1111/j.1365-2796.2004.01388.x

[26] Winblad B, Palmer K, Kivipelto M, Jelic V, Fratiglioni L, et al (2004) Mild cognitive impairment–beyond controversies, towards a consensus: report of the International Working Group on Mild Cognitive Impairment. J Intern Med 256: 240–246. 15324367 10.1111/j.1365-2796.2004.01380.x

[27] Roman GC, Tatemichi TK, Erkinjuntti T, Cummings JL, Masdeu JC, et al (1993) Vascular dementia: diagnostic criteria for research studies. Report of the NINDS-AIREN International Workshop. Neurology 43: 250–260. 8094895 10.1212/wnl.43.2.250

[28] McKeith IG, Galasko D, Kosaka K, Perry EK, Dickson DW, et al (1996) Consensus guidelines for the clinical and pathologic diagnosis of dementia with Lewy bodies (DLB): report of the consortium on DLB international workshop. Neurology 47: 1113–1124. 8909416 10.1212/wnl.47.5.1113

[29] McKhann G, Drachman D, Folstein M, Katzman R, Price D, et al (1984) Clinical diagnosis of Alzheimer’s disease: report of the NINCDS-ADRDA Work Group under the auspices of Department of Health and Human Services Task Force on Alzheimer’s Disease. Neurology 34: 939–944. 6610841 10.1212/wnl.34.7.939

[30] Katz S, Downs TD, Cash HR, Grotz RC (1970) Progress in development of the index of ADL. Gerontologist 10: 20–30. 5420677 10.1093/geront/10.1_part_1.20

[31] Fillenbaum GG, Smyer MA (1981) The development, validity, and reliability of the OARS multidimensional functional assessment questionnaire. J Gerontol 36: 428–434. 7252074 10.1093/geronj/36.4.428

[32] Lord SR, Ward JA, Williams P (1996) Exercise effect on dynamic stability in older women: a randomized controlled trial. Arch Phys Med Rehabil 77: 232–236. 8600863 10.1016/s0003-9993(96)90103-3

[33] Gnjidic D, Hilmer SN, Blyth FM, Naganathan V, Cumming RG, et al (2012) High risk prescribing and incidence of frailty among older community-dwelling men. Clin Pharmacol Ther 91: 521–528. 22297385 10.1038/clpt.2011.258

[34] Australian Institute of Health and Welfare (AIHW) (2011) Residential aged care in Australia 2009–10: a statistical overview. Aged care statistics series no. 35. Cat. no. AGE 66. Canberra.

[35] McCallum J, Simons AL, Simons J, Friedlander Y (2005) Patterns and predictors of nursing home placement over 14 years: Dubbo study of elderly Australians. Aust J Ageing 24: 169–173.

[36] Luppa M, Luck T, Matschinger H, Konig HH, Riedel-Heller SG (2010) Predictors of nursing home admission of individuals without a dementia diagnosis before admission - results from the Leipzig Longitudinal Study of the Aged (LEILA 75+). BMC Health Services Research 10: 186. 20584341 10.1186/1472-6963-10-186 PMC2909999

[37] Bharucha AJ, Pandav R, Shen C, Dodge HH, Ganguli M (2004) Predictors of nursing facility admission: a 12-year epidemiological study in the United States. J Am Geriatr Soc 52: 434–439. 14962161 10.1111/j.1532-5415.2004.52118.x

[38] Plassman BL, Langa KM, Fisher GG, Heeringa SG, Weir DR, et al (2008) Prevalence of cognitive impairment without dementia in the United States. Ann Intern Med 148: 427–434. 18347351 10.7326/0003-4819-148-6-200803180-00005 PMC2670458

[39] Manly JJ, Tang MX, Schupf N, Stern Y, Vonsattel JP, et al (2008) Frequency and course of mild cognitive impairment in a multiethnic community. Ann Neurol 63: 494–506. 18300306 10.1002/ana.21326 PMC2375143

[40] Busse A, Angermeyer MC, Riedel-Heller SG (2006) Progression of mild cognitive impairment to dementia: a challenge to current thinking. Br J Psychiatry 189: 399–404. 17077428 10.1192/bjp.bp.105.014779

[41] Fischer P, Jungwirth S, Zehetmayer S, Weissgram S, Hoenigschnabl S, et al (2007) Conversion from subtypes of mild cognitive impairment to Alzheimer dementia. Neurology 68: 288–291. 17242334 10.1212/01.wnl.0000252358.03285.9d

[42] Guo M, Gao L, Zhang G, Li Y, Xu S, et al (2012) Prevalence of dementia and mild cognitive impairment in the elderly living in nursing and veteran care homes in Xi’an, China. J Neurol Sci 312: 39–44. 21893322 10.1016/j.jns.2011.08.026

[43] Chukwujama O, Gormley N (2011) Mild cognitive impairment. N Engl J Med 365: 1358; author reply 1358–1359. 10.1056/NEJMc1108238 21991975

[44] Cohen-Mansfield J, Wirtz PW (2011) Predictors of entry to the nursing home: does length of follow-up matter? Arch Geron Geriatr 53: 309–315. 10.1016/j.archger.2010.12.009 PMC3111754 21251719

[45] Grundy E, Jitlal M (2007) Socio-demographic variations in moves to institutional care 1991–2001: a record linkage study from England and Wales. Age Ageing 36: 424–430. 17548425 10.1093/ageing/afm067

[46] McCann M, Donnelly M, O’Reilly D (2011) Living arrangements, relationship to people in the household and admission to care homes for older people. Age Ageing 40: 358–363. 21427114 10.1093/ageing/afr031

[47] Cooper R, Kuh D, Cooper C, Gale CR, Lawlor DA, et al (2011) Objective measures of physical capability and subsequent health: a systematic review. Age Ageing 40: 14–23. 20843964 10.1093/ageing/afq117 PMC3000177

[48] Tinetti ME, Williams CS (1997) Falls, injuries due to falls, and the risk of admission to a nursing home. N Engl J Med 337: 1279–1284. 9345078 10.1056/NEJM199710303371806

[49] Dunn JE, Furner SE, Miles TP (1993) Do falls predict institutionalization in older persons? An analysis of data from the Longitudinal Study of Aging. J Aging Health 5: 194–207. 10125444 10.1177/089826439300500203

[50] Holroyd-Leduc JM, Mehta KM, Covinsky KE (2004) Urinary incontinence and its association with death, nursing home admission, and functional decline. J Am Geriatr Soc 52: 712–718. 15086650 10.1111/j.1532-5415.2004.52207.x

[51] Thom DH, Haan MN, Van Den Eeden SK (1997) Medically recognized urinary incontinence and risks of hospitalization, nursing home admission and mortality. Age Ageing 26: 367–374. 9351481 10.1093/ageing/26.5.367

[52] Yaffe K, Fox P, Newcomer R, Sands L, Lindquist K, et al (2002) Patient and caregiver characteristics and nursing home placement in patients with dementia. JAMA 287: 2090–2097. 11966383 10.1001/jama.287.16.2090

[53] Chan DC, Kasper JD, Black BS, Rabins PV (2003) Presence of behavioral and psychological symptoms predicts nursing home placement in community-dwelling elders with cognitive impairment in univariate but not multivariate analysis. J Gerontol A Biol Sci Med Sci 58: 548–554. 12807927 10.1093/gerona/58.6.m548

